# Fabrication and Piezoresistive/Piezoelectric Sensing Characteristics of Carbon Nanotube/PVA/Nano-ZnO Flexible Composite

**DOI:** 10.1038/s41598-020-65771-x

**Published:** 2020-06-01

**Authors:** Shuaichao Chen, Jianlin Luo, Xiaoli Wang, Qiuyi Li, Liucong Zhou, Chao Liu, Chao Feng

**Affiliations:** 1 School of Civil Engineering, Qingdao University of Technology, Qingdao, 266033 China; 2 Institute of Process Engineering, Chinese Academy of Science, Beijing, 100190 China; 3 Collaborative Innovation Center of Engineering Construction and Safety in Shandong Blue Economic Zone, Qingdao University of Technology, Qingdao, 266033 China; 4 School of Architecture Engineering, Qingdao Agricultural University, Qingdao, 266109 China; 5 Jiangsu Key Laboratory of Construction Materials, School of Materials Science and Engineering, Southeast University, Nanjing, 211189 China

**Keywords:** Sensors and biosensors, Carbon nanotubes and fullerenes

## Abstract

Flexible sensors with a high sensitivity and wide-frequency response are essential for structural health monitoring (SHM) while they are attached. Here, carbon nanotube (CNT) films doped with various PVA fractions (CNT/PVA) and ZnO nanowires (nano-ZnO) on zinc sheets were first fabricated by functionalized self-assembly and hydrothermal synthesis processes. A CNT/PVA/nano-ZnO flexible composite (CNT/PVA/ZnO) sandwiched with a zinc wafer was then prepared by the spin-coating method. The piezoresistive and/or piezoelectric capabilities of the CNT/PVA/ZnO composite were comprehensively investigated under cyclic bending and impact loading after it was firmly adhered to a substrate (polypropylene sheet or mortar plate). The results show that the piezoresistive sensitivity and linear stability of the CNT/PVA films doped with 20%, 50%, and 100% PVA during bending are 5.47%/mm, 11.082%/mm, and 11.95%/mm and 2.3%, 3.42%, and 4.78%, respectively. The piezoelectric sensitivity, linear stability, and response accuracy of the CNT/PVA/ZnO composite under impulse loading are 4.87 mV/lbf, 3.42%, and 1.496 ms, respectively. These merits support the use of CNT/PVA/ZnO as a piezoresistive and/or piezoelectric compound sensor to monitor the static/dynamic loads on concrete structures while it is attached.

## Introduction

Structural health monitoring (SHM) of the structural integrity of concrete structures under external static/dynamic loading has become an important tool to ensure the safety of various types of infrastructure. SHM aims to design, verify and optimize structures and also provide real-time structural damage detection and assessment by comprehensively utilizing signal processing, sensing, communications, and other cross-subject technologies. Sensors with accurate and fast-response sensing capabilities are fundamental and essential to SHM, and sensor development has attracted considerable worldwide attention^1^. Over the last several decades, fiber Bragg grating (FBG) sensors have increasingly been developed for SHM owing to their advantages of high linearity, accurate sensing, corrosion resistance, small size, and long-distance distribution^2^. However, FBG has some disadvantages that restrict its wider application in SHM, such as high brittleness if there is no packaging process, incompatibility when embedded in concrete structures, and sophisticated synthesis, substrate selection and packaging^3,4^ processes.; in addition, the FBG needs to be equipped with a signal demodulator^5^. Recently, Chung *et al*.^6^, Sun *et al*.^7^, Li *et al*.^8^, Han *et al*.^9^, Luo *et al*.^10^, Dong *et al*.^11^, and Huang *et al*.^12^ introduced conductive fillers, such as steel fibers, carbon fibers, carbon black, nickel powder, carbon nanotubes, carbon nanofibers, and lead zirconium titanate powder, into cement/mortar to develop intrinsic piezoresistive or piezoelectric sensors for the SHM of concrete structures and achieved good compatibility, a relatively high sensitivity, and high linearity. However, most of the abovementioned sensors did not necessarily have the capability of simultaneously detecting static or dynamic signals, which have indeed been frequently encountered during SHM under cyclic or impulse loading^13,14^. Moreover, because flexibility, good adhesion to the substrate interface, and an arbitrary arrangement and distribution are measurement requirements, flexible sensors have also achieved intensive use in SHM applications. Flexible electrical strain gauges have been widely developed due to their low cost and relative sensing stability, but these strain gauges also have unavoidable drawbacks, such as low sensitivity, low resolution and high energy consumption^15,16^. Flexible sensors with higher sensitivities and wider amplitudes are desirable for SHM^17^.

Since being reported in 1991, carbon nanotubes (CNTs) with a high specific surface area, good mechanical strength, and excellent conductivity have been developed into various types of flexible sensors by different treatment processes^18,19^. Cai *et al*. reported carbon nanotube (CNT)-based capacitive strain sensors that can detect strains up to 300% with excellent durability even after thousands of cycles^19^. Cao *et al*. prepared a multiwall CNT (MWNT) piezoresistive film in aqueous solution, directly glued it on a stainless-steel substrate and studied the temperature-dependent piezoresistive effect of the MWNT films. The results showed that with increasing temperature from 15 °C to 50 °C, the gauge factor increased from 46 to 58.6 for the pristine MWNT films and from 75.4 to 91.2 for the chemically treated MWNT films, respectively, under a microstrain of 500^20^. Gau *et al*. developed a polyimide-CNT film by the spin coating method and formed it into a flexible sensor for structural pressure detection and found that the polyimide-CNT sensor had the basic sensing characteristics of a sensor, such as sensitivity, linearity and repeatability^21^. Alexopoulos *et al*. developed embedded PVA-CNT fiber material and used it as a strain sensor for damage monitoring and found that the effect of prestretching enabled the PVA-CNT fiber material to be used as a strain sensor, and the fibers did not leave a residual resistance after each unloading^22^. Maddipatla *et al*. dipped a novel CNT-based printed conductive ink onto a PDMS substrate, produced a flexible pressure sensor, and observed that this CNT-based pressure sensor produced an 8.2% change in the capacitance when there was a maximum detectable pressure of 337 kPa^23^. Loh *et al*. prepared a CNT piezoresistive film through a layer-by-layer self-assembly process^24^. However, the above flexible CNT-based sensor only had piezoresistive behavior for sensing static signals but could not effectively meet the SHM requirement of simultaneously sensing static and dynamic signals.

ZnO possesses a hexagonal single-crystal wurtzite structure, and its maximal piezoelectric coefficient (*d*_33_) and piezoelectric electromotive force can reach 12.3 pC/N and more than 100 mV, respectively. In fact, a ZnO piezoelectric pressure sensor has the advantages of easy integration and high sensitivity, for example^25^, but a conventional tablet-type ZnO piezoelectric wafer has the disadvantages of high brittleness, irregular arrangement of crystal grains, and incompatibility with a flexible film. The utilization of nano-ZnO grains with a regular lattice arrangement is an efficient way to avoid the above problems, and a substrate can be pretreated by a spin coating process to form nano-ZnO nuclei that provide a template for the growth of nano-ZnO grains^26^. At present, liquid phase synthesis is the mainstream method for the preparation of nano-ZnO, which has the advantages of uniform particle distribution, controllable growth direction and mild processing environment^27^. Yin *et al*. synthesized ZnO nanorods by a low-temperature solvothermal method and found that the aspect ratio of the ZnO nanorods increased with an increase in the concentration ratio of [OH^-^] to [Zn^2+^]^28^. Shao *et al*. prepared films comprising ZnO nanorod arrays on silicon substrates by a hydrothermal method, and the electrical characteristics of a Pt Schottky contact with ZnO nanorods were studied with atomic force microscopy. An ideality factor of 3.1 was derived from the current-voltage characteristic curve of the junction^29^. Parangusan *et al*. proposed a free-carrier-modulated ZnO:nitrogen thin film-based flexible nanogenerators (NZTF-FNGs). Its output voltage (peak-to-peak) on average reached 0.3 V, which was much higher than that of conventional ZTF-FNGs that reached only 0.004 V. The nano-ZnO array synthesized by the hydrothermal method achieved a satisfactory response in detecting the piezoelectric voltage.

On the one hand, PVA is an electrolyte with excellent adhesion, and doped PVA could improve the ductility and flexible integrity of self-assembled CNT films^30^. On the other hand, ZnO nanocrystal grains can be arranged in an orderly manner to obtain a nano-ZnO array; therefore, its mechanoelectrical response capability can be improved^31^. Thus, we proposed a type of sandwich film sensor composed of a CNT/PVA film and nano-ZnO array that served as piezoresistive and piezoelectric components, respectively. In particular, the CNTs were first positively and negatively charged by the mixed acid oxidation and cyano reduction methods, respectively, and the CNT/PVA films were fabricated from CNT films by the self-assembly method along with doping with PVA. A ZnO nanowire (nano-ZnO) array was grown on one side of the zinc substrate by the hydrothermal method. A sandwich-like CNT/PVA/ZnO flexible composite was fabricated via a spin coating process after the CNT/PVA layer was coated on the other side of the zinc substrate. The piezoresistive and piezoelectric capabilities of the CNT/PVA film and CNT/PVA/ZnO flexible composite under cyclic bending and impact loading were comprehensively characterized. Herein, the sensor characteristics of two types of films were also investigated and compared to develop a novel sensor with high sensitivity and wide frequency response for SHM.

## Experimental details

### Raw materials

Multiwall CNTs were purchased from Chengdu Organic Chemical Co. Ltd., Chinese Academy of Sciences (Chengdu, China), and the main physical properties and corresponding providers of the other chemical reagents used in this study are listed in the supplementary information.

### Synthesis of nano-ZnO

**Figure 1:**
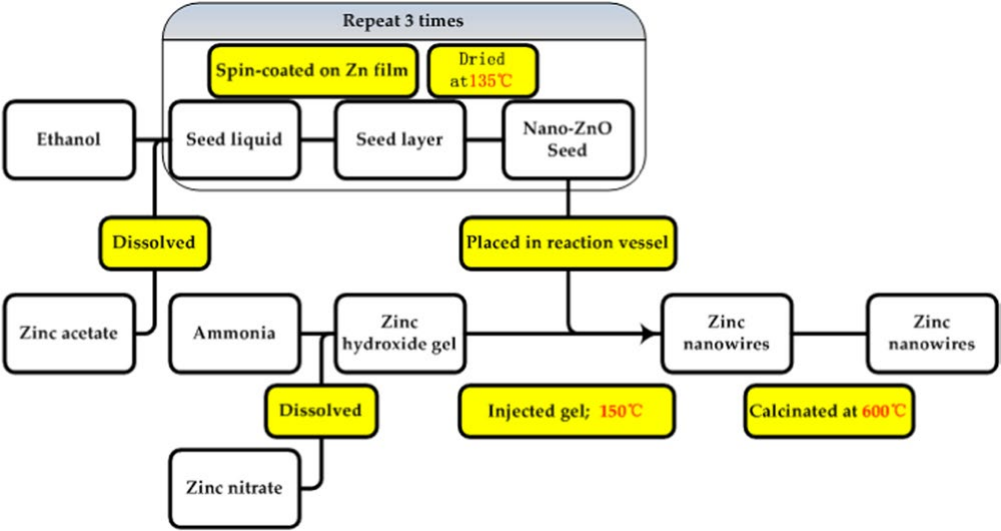
Schematic procedure for nano-ZnO array synthesis on one side of the zinc substrate.

The nano-ZnO array was synthesized by the hydrothermal method in accordance with ref. ^31^, and the corresponding procedure for nano-ZnO synthesis on a zinc substrate is schematically demonstrated in Fig. 1. The nano-ZnO array was quenched in a muffle furnace (XZK-3 type, Longkou, China) with a heating rate of 20 °C/min at 600 °C for 30 min, and the other side of the zinc substrate sheet was polished before coating the self-assembled CNT film.

### Fabrication of the self-assembled CNT/PVA film

**Figure 2:**
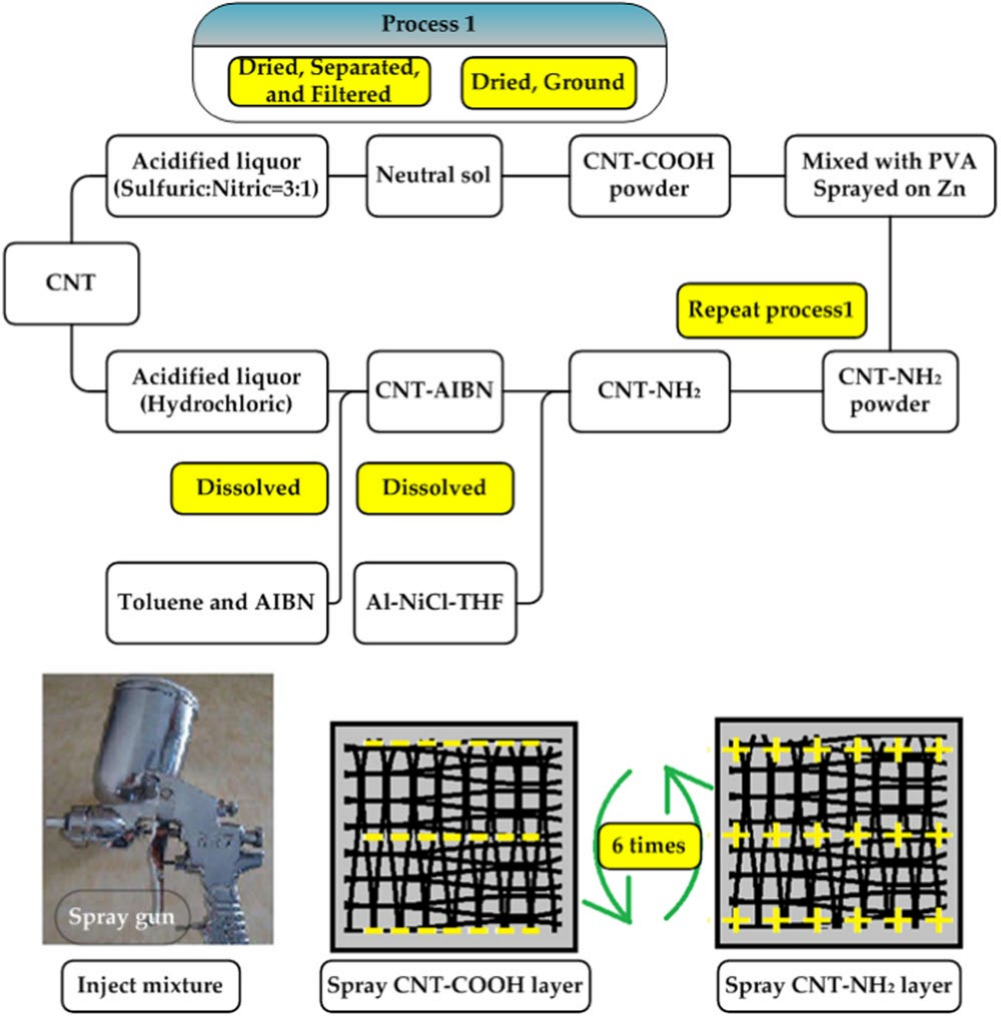
Schematic diagram for carboxylation, amination preparation of the CNTs, and self-assembly of the CNT/PVA film.

The processes for the positive and negative functionalization of the CNTs (CNT-COOH and CNT-NH_2_) and self-assembled CNT films were similar to those in ref. ^32^. First, 1 mg/ml CNT-NH_2_ and 1 mg/ml CNT-COOH suspensions were ultrasonically treated for 2 h at 40 kHz (KH5500H type, Changzhou, China). After 24 h incubation, the above CNT-NH_2_ and CNT-COOH suspensions were doped with PVA solutions with mass ratios of the PVA solution to the CNT-NH_2_ or CNT-COOH suspensions of 0.2:1, 0.5:1, and 1:1, and then the suspensions were bath treated at 80 °C for 2 h. The CNT-COOH/PVA suspension was first sprayed on the polished surface of the opposite side of the zinc substrate by a sprayer (WA-101 type, Hangzhou, China). Then, the suspension was deposited by a spin-coater (KW-4B type, Beijing SETCAS Electronic Co. Ltd., China) with low and high speeds of 500 rpm and 1500 rpm, respectively, for 60 s. After drying at 80 °C for 15 min in a vacuum oven (DZF-6210 type, Shanghai, China), a CNT-NH_2_/PVA suspension was sprayed on the first CNT-COOH/PVA layer and spin-coated, and one CNT-COOH/CNT-NH_2_/PVA double layer was finally formed after drying at 80 °C for another 15 min. The above processes were repeated 6 times, and the CNT/PVA film with six double layers was achieved after drying at 80 °C for 30 min. The procedure for the preparation of the CNT-COOH, CNT-NH_2_ and self-assembled CNT/PVA film is schematically demonstrated in Fig. 2.

### Fabrication of CNT/PVA/ZnO flexible sandwich composite

**Figure 3:**
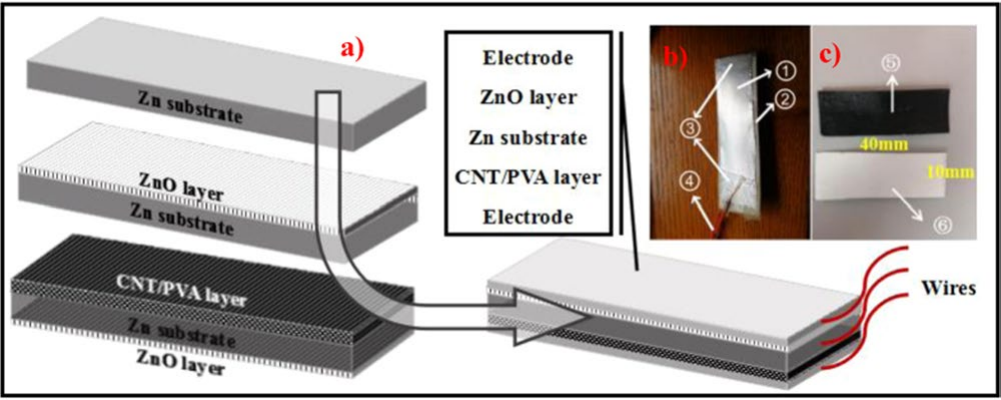
(**a**) Schematic diagram of the CNT/PVA/ZnO composite assembly process, (**b**) configuration of the CNT/PVA/ZnO, and (**c**) CNT/PVA film from the front and nano-ZnO array from the front (① silver electrode, ② ③ adhesive tape, ④ wires, ⑤ CNT/PVA layer, and ⑥ nano-ZnO layer.

First, the nano-ZnO array and CNT/PVA film layer in the CNT/PVA/ZnO flexible sandwich composite were sequentially synthesized in accordance with the abovementioned methods in Sections 2.2 and 2.3, respectively, where a schematic of the fabrication process is shown in Fig. 3a. Second, the electrodes and wires were connected surfaces of the CNT/PVA/ZnO flexible composite. Third, adhesive tape was precisely placed on the edge of the zinc substrate so as to avoid a possible short circuit between the electrodes and the substrate and the fixed wire. A schematic of the configuration is shown in Fig. 3b.

### Characterization methods

Scanning electron microscopy (SEM, S3500N type, Hitachi Corp., Japan) was used to observe the morphology and microstructures of the CNT/PVA film, nano-ZnO array, and CNT/PVA/ZnO flexible composite. A quasi-static *d*_33_/*d*_31_ measuring instrument (ZJ-6A type, Institute of Acoustics, Chinese Academy of Sciences, China) was employed to measure the *d*_33_ of the nano-ZnO sheet after cutting it into 10 mm × 10 mm × 0.4 mm samples where the size factor is 1.

The piezoresistive and piezoelectric sensing characteristics of the CNT/PVA/ZnO flexible composite were tested by cyclic three-point bending loads and impulse load testing methods, respectively, and the details are demonstrated in supplementary information.

As revealed in Figure S2 from supplementary information, the CNT/PVA/ZnO layer specimen was attached onto a cantilever. Different signal channels of DASP V11 data acquisition system were separately collected the signals from the nano-ZnO and the CNT/PVA layers, with CNT/PVA, nano-ZnO layer respectively acting as a piezoresistive resistor, and a piezoelectric capacitor. The acceleration signals from the nano-ZnO thin layer mainly reflected the dynamic or impact loads, while the voltage signals from CNT/PVA layer could sense the static and quasi-static loads aiding with 2 V DC power supply. It benefits for synchronous and simultaneous detections to the complicate loads in SHM, effectively avoids drawbacks while using traditional either piezoresistive or piezoelectric sensor.

## Results and discussion

### Surface morphology and SEM analysis

**Figure 4:**
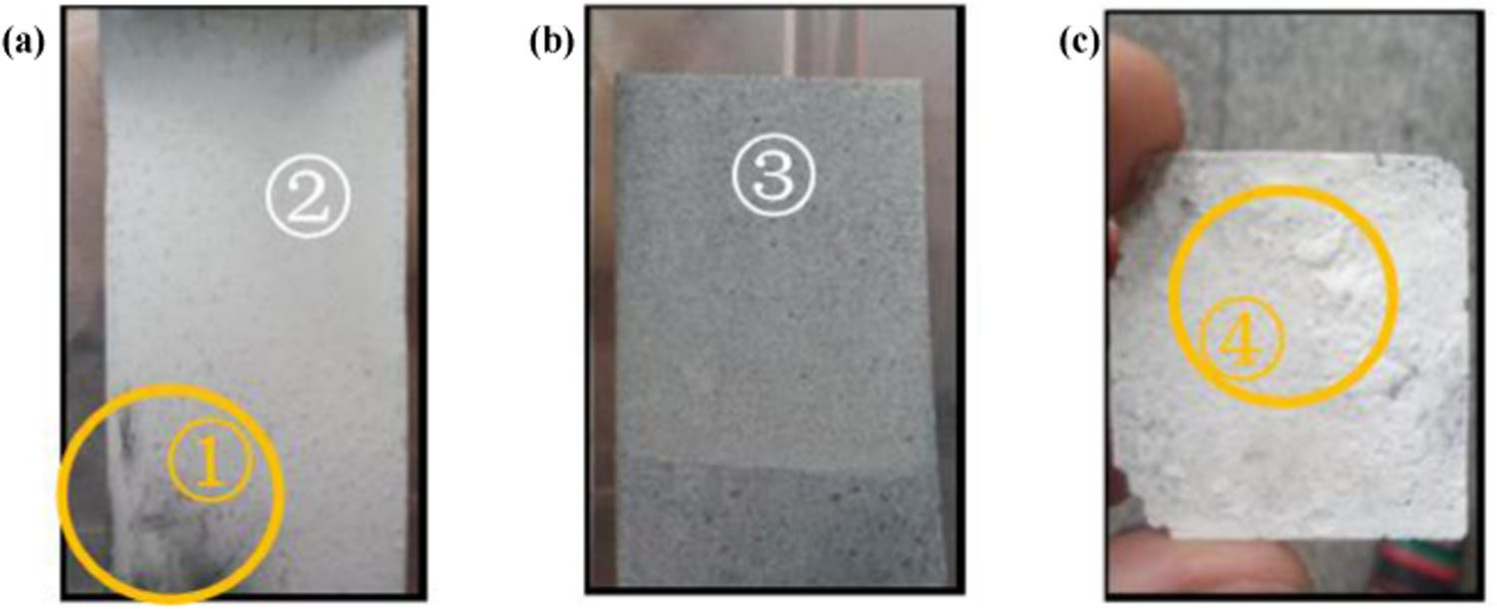
Surface morphologies of the nano-ZnO flakes after hydrothermal processing with different *w*_*zn2+*_ values: (**a**) 1 × 10^−5^ mol/L, (**b**) 2 × 10^−5^ mol/L, and (**c**) 10 × 10^−5^ mol/L (① uncovered substrate, ② bright white region, ③ yellowish gray region, and ④ grainy and pale yellow region).

Figure 4 presents the surface appearance of the nano-ZnO grown on a zinc substrate in various zinc ion molar concentrations (*w*_*zn2+*_). When the *w*_*zn2+*_ is 10^−5^ mol/L, the surface appears bright white and there is an uncovered area of substrate in the left corner (circled in Fig. 4a)), which implies that the nano-ZnO layer is extremely thin, and as *w*_*zn2+*_ increases to 2 × 10^−5^ mol/L, the surface color gradually changes to yellowish gray. When *w*_*zn2+*_ reaches 10 × 10^−5^ mol/L, the surface is no longer smooth, has a grainy appearance (circle in Fig. 4c)), and the color changes to pale yellow.

**Figure 5:**
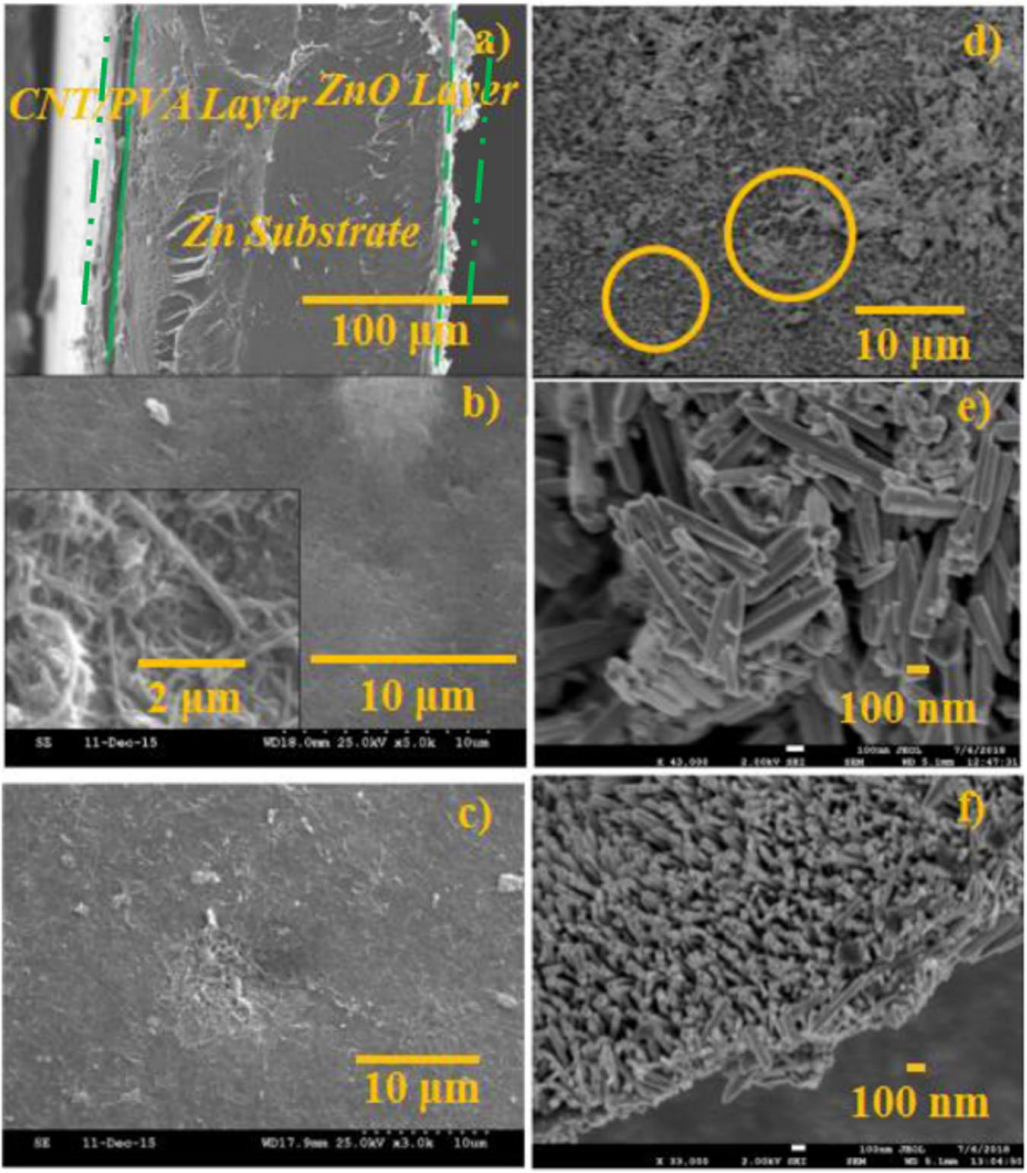
SEM images of the CNT/PVA/ZnO composite with *m*(CNT):*m*(PVA) of 0.5 and *w*_*zn2+*_ of 2 × 10^−5^ mol/L: (**a**) overall array appearance of the CNT/PVA/ZnO, (**b**) overall morphology of the CNT/PVA film with inset of magnified morphology of the CNTs (×5.0k), (**c**) overall morphology of the CNT/PVA film with CNT clumps (×3k), (**d**) overall morphology of the nano-ZnO array (×2.0k), (**e**) high-magnification image of the morphology of the large-sized nano-ZnO with disorganized crystals (×43k), and (**f**) high-magnification image of the morphology of the small-sized nano-ZnO array with a vertical crystal arrangement (×33k).

Figure 5 shows the overall and side views of the CNT/PVA/ZnO flexible composite. The images show an obvious interface between both film layers, and the total width of the flexible composite along with the zinc substrate is approximately 200–300 μm, where the thickness of the CNT/PVA film layer, nano-ZnO layer, and substrate is approximately 30–40 μm, 10–20 μm, and 200 μm, respectively. The CNT/PVA layer on the left side of the zinc substrate has a smooth facade, while the nano-ZnO layer on the right side is uneven, and the different morphologies show a good interface contact. The CNT/PVA film surface is smooth with a few light spots, and its appearance is attributed to a uniform dispersion of the CNTs, as shown in Fig. 5b.

The particle size and crystal arrangement of the nano-ZnO determines the apparent color, as revealed in Fig. 5d. A large number of nano-ZnO floccules is deposited; the nano-ZnO crystal arrangement is disorganized, and the mean particle size is over 1 μm, which means that this type of nano-ZnO experiences optical absorption readily compared with that of the nano-ZnO array with a particle size below 100 nm (Fig. 5e). It can be concluded that the larger the particle size is, the more yellow the material becomes as the accumulation of the nano-ZnO increases. Simultaneously, the electrical signal of the corresponding nano-ZnO film is not obvious when subjected to external pressure due to this kind of disordered aggregation and agglomeration of the nano-ZnO (Fig. 5e).

As demonstrated in Fig. 5c, a homogeneous self-assembled CNT system with a high dose and good dispersion can be achieved with a uniform distribution of functionalized CNT-COOH and CNT-NH_2_ even though a minor amount of accumulation and agglomeration still exists. As shown in Fig. 5d, the patterns of the nano-ZnO array are regular hexagonal pyramids and hexagonal prisms^33,34^ with lengths and diameters less than 1 μm and 0.1 μm, respectively. Notably, a minor amount of flocculent aggregates grew on the nano-ZnO array owing to the aggregation of ZnO colloids, as shown in Fig. 5e^35^. In comparison, Fig. 5f presents a relatively uniform nano-ZnO array whose growth direction is vertical to the zinc substrate. Indeed, the preformed seed layer provides crystal nuclei for the formation of ZnO crystals, inducing the ZnO to grow vertically from the seed layer, thereby forming a crystalline nano-ZnO array, as shown in Fig. 5f. However, ZnO, which is far from the seed layer in the dispersed colloidal system, spontaneously forms a unit cell until it develops into large crystalline grains, causing nano-ZnO deposition on the seed layer by overcoming the buoyancy of the liquid. The neat arrangement and organized array formation allow the nano-ZnO arrays to produce a uniform charge distribution, and the electrical signal increases when subjected to a dynamic load.

### Piezoresistivity of the CNT/PVA/ZnO flexible composite

#### Cyclic three-point bending test

**Figure 6:**
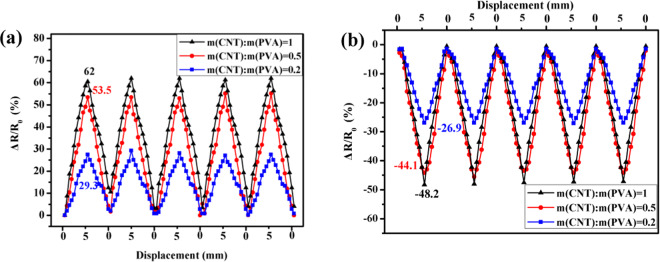
Relationship between Δ*R*/*R*_0_-*X* for the CNT/PVA/ZnO under cyclic loading on the: (**a**) tension side and (**b**) compression side.

Figure 6 shows the relationship between *ΔR* and the cyclic displacement of the CNT/PVA/ZnO composite.

As shown in Fig. 6, at the mid-span on the tensile side, the dose of PVA can significantly affect *R* and *ΔR*, and the *ΔR/R*_*0*_ peak of the CNT/PVA/ZnO film is approximately 29%, 54%, and 62% when *m*(CNT):*m*(PVA) is 0.2, 0.5, and 1, respectively. When *m*(CNT):*m*(PVA) is 1, the highest *ΔR/R*_*0*_ amplitude of the CNT/PVA/ZnO film among the samples herein is achieved. At the mid-span on the compressed side, the *ΔR/R*_*0*_ peak is approximately 27%, 44%, and 48% when *m*(CNT):*m*(PVA) is 0.2, 0.5, and 1, respectively, and the highest *ΔR/R*_*0*_ amplitude can also be found when *m*(CNT):*m*(PVA) is 1. It is worth pointing out that the *ΔR/R*_*0*_ amplitude when *m*(CNT):*m*(PVA) is 0.5 is close to that when *m*(CNT):*m*(PVA) is 1 in either tension or compression mode; there is also good repeatability, which means the CNT/PVA film with *m*(CNT):*m*(PVA) of 0.5 is the most cost-effective among the samples herein. Loh *et al*. documented that his self-assembled CNT films had a piezoresistive sensitivity ranging from 5%/mm~20%/mm^33^; however, the fluctuation of the piezoresistive sensitivity of his sensor was higher than ours, which was a drawback for its potential SHM applications.

The *R* and Δ*R* of the CNT/PVA component of the CNT/PVA/ZnO film both increase as the tension increases because the connecting pathways among the conductive but well-distributed CNTs are accordingly decreased or the barrier widths between the CNTs are increased, which dramatically reduces the current densities of the electrons inside the CNTs; therefore, Δ*R* has the highest value when the displacement (tension) reaches its highest value. Conversely, the connecting pathways among the CNTs are increased, or the barrier widths between CNTs are decreased on the compression side, and Δ*R* accordingly has the lowest value when the displacement (compression) reaches its highest value^36,37^.

#### Sensitivity, linearity and repeatability of the CNT/PVA/ZnO flexible composite

The sensitivity (*S*_e_) of the CNT/PVA/ZnO flexible composite under cyclic bending and impulse loading can be calculated by the following formula^38,39^:

1 $${S}_{e}=\frac{\varDelta R/{R}_{0}}{\varepsilon }$$

where *ε* = Δ*L*/*L*_0_, Δ*L* is the change in length of the film, *L*_*0*_ is the initial length of the film, and Δ*R*/*R*_0_ is the resistance change rate vs. initial *R* (*R*_0_).

As revealed in Table 1, the *S*_*e*_ of each sample in the same group is close and has a low deviation, which indicates that each group of CNT/PVA/ZnO flexible composites possesses excellent repeatability. The mean *S*_*e*_ of the CNT/PVA/ZnO flexible composite is 5.47%/mm, 11.08%/mm, and 11.95%/mm when *m*(CNT):*m*(PVA) is 0.2, 0.5, and 1, respectively, under tension, and the value of the mean *S*_*e*_ is 5.607%/mm, 9.74%/mm, and 9.78%/mm under compression, respectively.

**Table 1:** Sensitivity (*S*_e_) of CNT/PVA/ZnO with various PVA doping amounts (Unit is %/mm).

*m*(CNT):*m*(PVA)	0.2		0.5		1	
*S*_e_	Tens. side	Comp. side	Tens. side	Comp. side	Tens. side	Comp. side
Sample 1	5.448	5.595	11.102	9.762	11.957	9.787
Sample 2	5.461	5.608	11.02	9.68	11.951	9.781
Sample 3	5.5	5.617	11.118	9.778	11.941	9.771
Mean & deviation	5.47 ± 0.03	5.61 ± 0.01	11.08 ± 0.04	9.74 ± 0.04	11.95 ± 0.01	9.78 ± 0.01

The linearity (*e*_*l*_) of the CNT/PVA/ZnO composite under cyclic loading can be calculated by the following formula^38,39^:

2 $${e}_{l}=\pm \frac{\varDelta max}{{(\Delta R/{R}_{0})}_{F\bullet S}}$$

where ∆max and (*ΔR/R*_*0*_)_*F•S*_ represent the maximum deviation between the *ΔR/R*_*0*_-*X* curve and the linear fitting line and the full-scale output of *ΔR/R*_*0*_, respectively.

**Figure 7:**
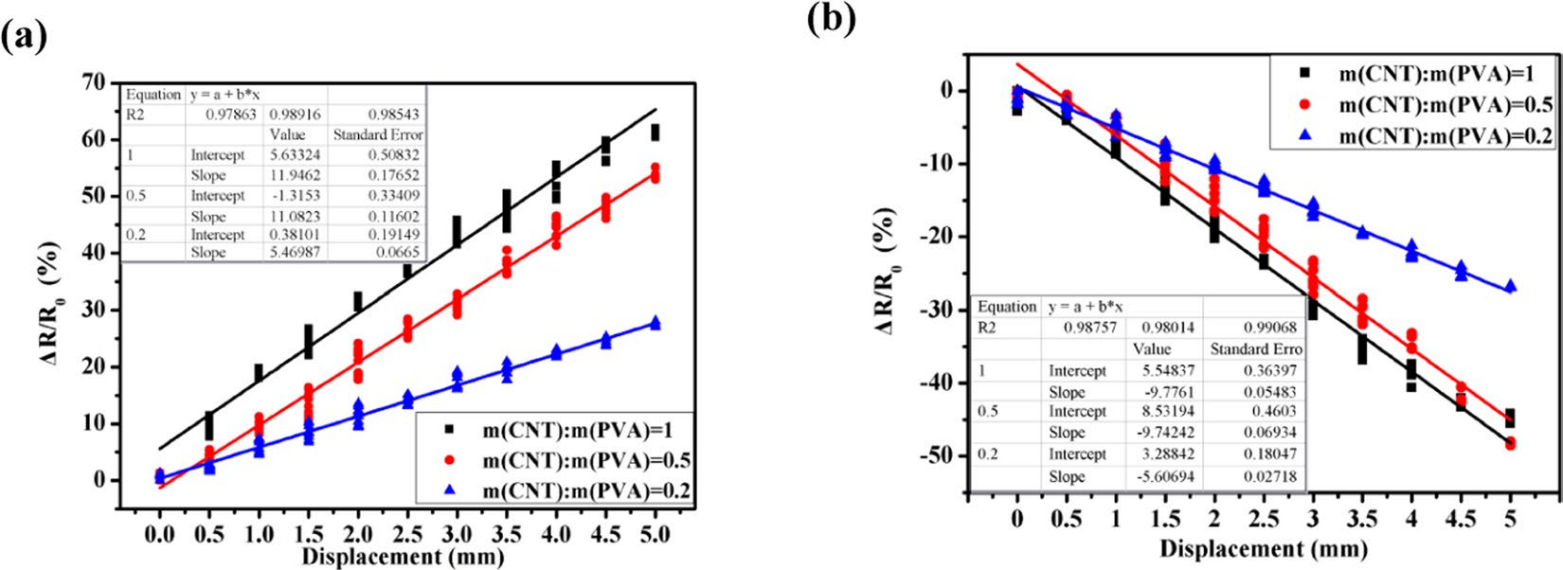
Regression fitting with *ΔR/R*_*0*_-*X* for the CNT/PVA/ZnO composites under bending with various PVA doping amounts on the: (**a**) tension side and (**b**) compression side.

Figure 7 shows the *e*_*l*_s of the CNT/PVA/ZnO composite with *m*(CNT):*m*(PVA) values of 0.2, 0.5, and 1 under cyclic loading; the regressed linear equations are presented in Table 2 with correlation coefficients of 0.98543, 0.98976, and 0.97863 on the tension side and 0.99068, 0.98014 and 0.98757 on the compression side, respectively.

**Table 2:** Regressed linear equations for the *Se*s of the CNT/PVA/ZnO composites with various PVA doping amounts.

*m*(CNT):*m*(PVA)	Tension side	Compression side
0.2	$y=5.47x+0.381\ast$	$y=-\,5.607x+0.485$
0.5	..	..
1.0	$y=11.95x+5.633$	$y=-\,9.78x+0.6603$

*Note that *y* and *x* represent the fitted *ΔR/R*_*0*_ and *ɛ* of the *ΔR/R*_*0*_ − *ɛ* curve under tension and compression, respectively.

**Figure 8:**
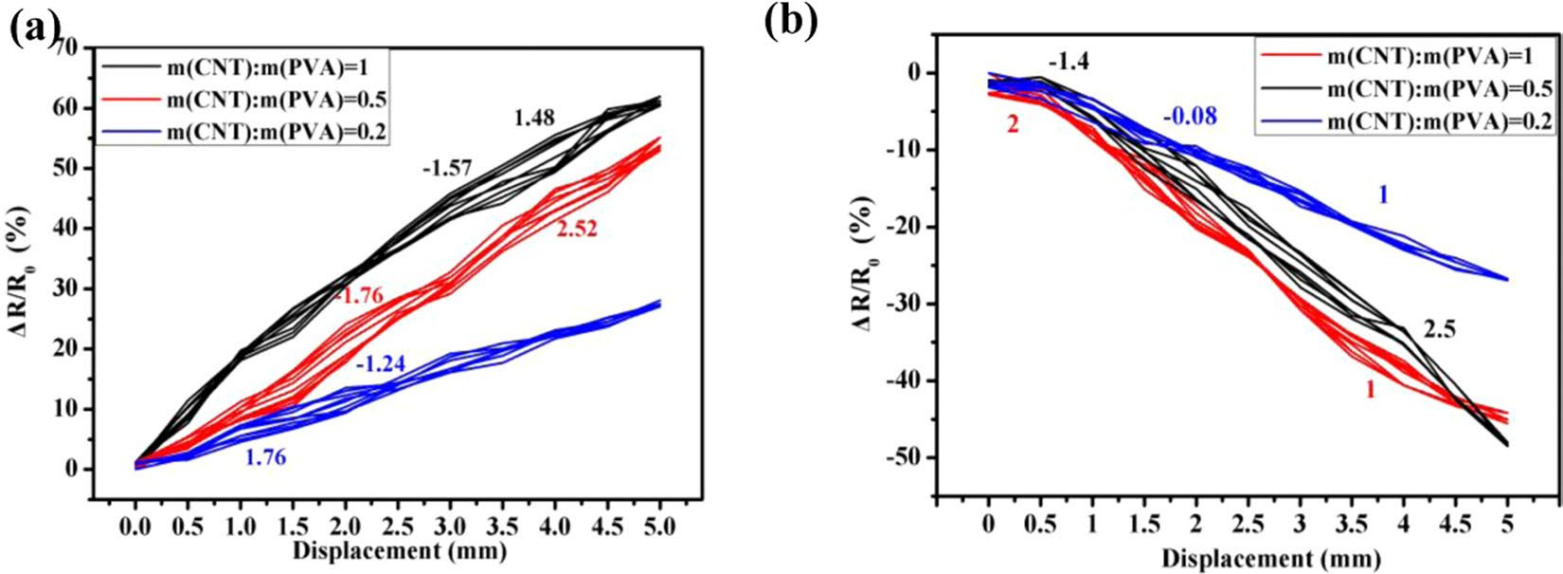
Relationship between *ΔR/R*_*0*_-*X* for the CNT/PVA/ZnO with various PVA doping amounts under bending on the: (**a**) tension side and (**b**) compression side.

Fig. 8 presents the *ΔR/R*_*0*_-*X* relationship of the CNT/PVA/ZnO composites with various PVA doping amounts under a bending load. Their *e*_l_s values are shown in Table 3. The *e*_*l*_ value for each sample in the same group is close and has a low deviation. Furthermore, the *e*_*l*_s of the CNT/PVA/ZnO composites with *m*(CNT):*m*(PVA) values of 0.2, 0.5, and 1 are 2.30%, 3.42%, and 4.78% on the tension side, respectively, whereas they are 2.38%, 2.88%, and 4.92% on the compression side, respectively. These results imply that the *e*_l_s of all groups of the CNT/PVA/ZnO composites are less than 5%, showing a good and stable linearity.

**Table 3:** Linear stabilities (e_*l*_s) of the CNT/PVA/ZnO with various PVA doping amounts (Unit is %).

*m*(CNT):*m*(PVA)	0.2		0.5		1	
e_*l*_	Tens. side	Comp. side	Tens. side	Comp. side	Tens. side	Comp. side
Sample 1	2.23	2.28	3.35	2.85	4.79	4.78
Sample 2	2.26	2.33	3.39	2.7	4.81	4.91
Sample 3	2.41	2.53	3.52	3.09	4.74	5.07
Mean & deviation	2.30 ± 0.10	2.38 ± 0.15	3.42 ± 0.01	2.88 ± 0.20	4.78 ± 0.05	4.92 ± 0.15

The repeatability (*r*_*e*_) of the CNT/PVA/ZnO composites under cyclic loading can be calculated by the following formula^38,39^:

3 $${r}_{e}=\pm \frac{1}{2}\frac{{\varDelta }_{{ma}{{x}}_{1}}}{\varDelta R/{R}_{0}}\times 100 \% $$

where ∆max1 and (*ΔR/R*_*0*_)_*F•S*_ represent the maximum output nonrepetitive error and full-scale output of *ΔR/R*_*0*_, respectively.

As shown in Table 4, the *r*_*e*_ values of the CNT/PVA/ZnO composite with *m*(CNT):*m*(PVA) values of 0.2, 0.5, and 1 are 3.0%, 2.36% and 1.27% on the tension side, respectively, whereas they are 1.86%, 2.27%, and 2.59% on the compression side, respectively. All *r*_*e*_s are below 5%, which indicates good sensing stability for the flexible composite^40–43^. The data show that the linearity index between individuals is not very volatile and has a high similarity.

**Table 4:** Repeatability (*r*_*e*_) for CNT/PVA/ZnO with various PVA doping amounts (Unit is %).

m(CNT):m(PVA)	0.2		0.5		1	
*r*_*e*_	Tens. side	Comp. side	Tens. side	Comp. side	Tens. side	Comp. side
Sample 1	2.93	1.84	2.41	2.42	1.38	2.51
Sample 2	2.96	1.77	2.18	2.31	1.21	2.69
Sample 3	3.11	1.97	2.49	2.08	1.22	2.57
Mean & deviation	3.0±0.03	1.86 ± 0.03	2.36 ± 0.03	2.27 ± 0.03	1.27 ± 0.03	2.59 ± 0.03

As revealed in Table 5, excellent sensing parameters and stable linear responses render our CNT/PVA/ZnO composite feasible as a stress/strain sensor. An excessively high sensitivity results in failure during large deformation of the structure, and a sensitivity that is too low greatly increases the monitoring difficulty of the system^44^. The CNT/PVA/ZnO composite in our study and ref. ^43^ both show high sensitivity, but the linearity and repeatability in this study are superior to that in the cited reference.

**Table 5:** Piezoresistive sensing parameters of CNT/PVA/ZnO under cyclic bend loading compared with those of others.

	Linearity *e*_*l*_ (%)	Sensitivity *S*_e_ (mV/N)	Repeatable stability *r*_*e*_ (%)
This work	4.78	12.69	1.27
ref. ^39^	1.5	4.45	—
ref. ^40^	0.988	744	—
ref. ^41^	2.4	2.0	—
ref. ^42^	6.43	42.71	2.06

### Piezoelectric/piezoresistive properties of CNT/PVA/ZnO sheets under impulse loading

**Figure 9:**
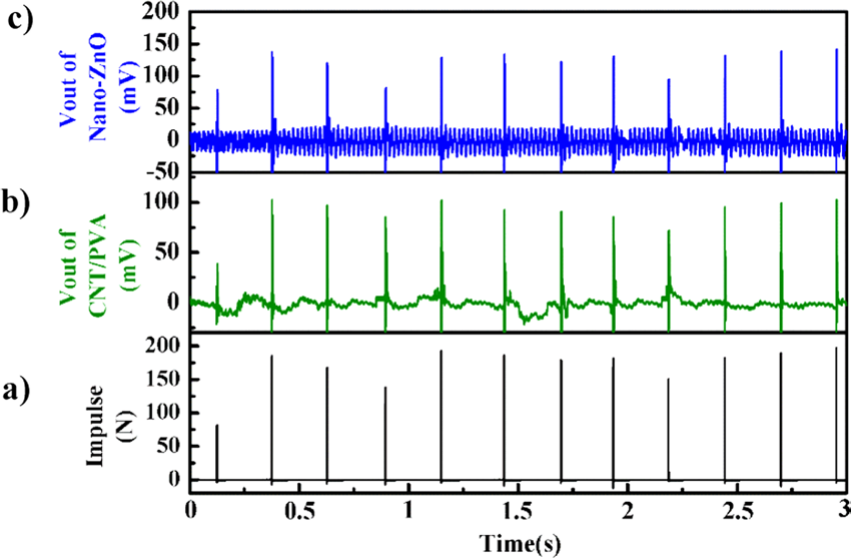
Voltage output history of the CNT/PVA/ZnO sheet under impulse loading: (**a**) force of the hammer, (**b**) the CNT/PVA film, and (**c**) the nano-ZnO film.

**Figure 10:**
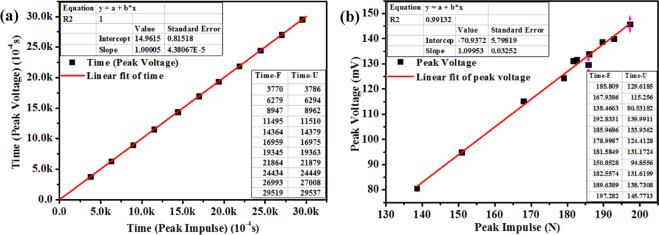
Fitting line for the (**a**) voltage peak-impulse peak along with time for the nano-ZnO component and (**b**) voltage peak along with the impulse level.

Figure 9 shows the vibration history after 12 impulses of the hammer within 3 s on the CNT/PVA/ZnO sheet. First, we observe the degree of coincidence at the responding time node, as shown in Fig. 10a, after which we convert the relationship between the load and the electrical signal only if the time node is matched (Fig. 10b). Figure 9 shows data points for the impulse voltage peaks that correspond to their impulse load peaks along with their response time, and the correlation of these sample points for the voltage and load peaks along with the response time is calculated. The equation for the linear fit is ${y}_{2}=1.00005{x}_{2}+14.96156$, the correlation coefficient *R*^2^ is 1.0 and the response time is 1.496 ms; also, *y*_2_, *y*_4_, and *x*_2_, *x*_4_ represent the time of the peak impulse and time of peak the voltage, respectively, and *y*_3_, *y*_5_, and *x*_3_, *x*_5_ represent the peak voltage and peak impulse, respectively.

In Fig. 10b, the voltage fitting line is ${y}_{3}=1.09953{x}_{3}-70.9372$ with an *R*^2^ of 0.99132, a sensitivity of 4.87 mV/lbf, and a maximum linearity error of 5.7%. It can be seen from *R*^2^ that the peak linearity is improved; that is, the piezoelectric response capability can meet the demand. The sensitivity is compared with piezoelectric sensor products produced in America by DYTRAN^45^, and the model and relative parameters are shown in Table 6. Our sandwich sensor is suitable as a piezoelectric sensor to detect impulse loads (4.93 × 10^5^ Pa).

**Table 6:** Comparison of piezoelectric parameters from our sensor and those of IEPE sensors under impulse loading.

Sensor type	Sensitivity		Sensing amplitude (Pa)
	(mV/N)	(mV/lbf)	
This work	1.0995	4.87	4.93 × 10^5^
Force Sensors, IEPE 1203v2	2.25	10.0	5 × 10^2^
Force Sensors, IEPE 1203v3	1.12	5.0	1 × 10^3^
Force Sensors, IEPE 1203v4	0.225	1.0	5 × 10^3^

**Figure 11:**
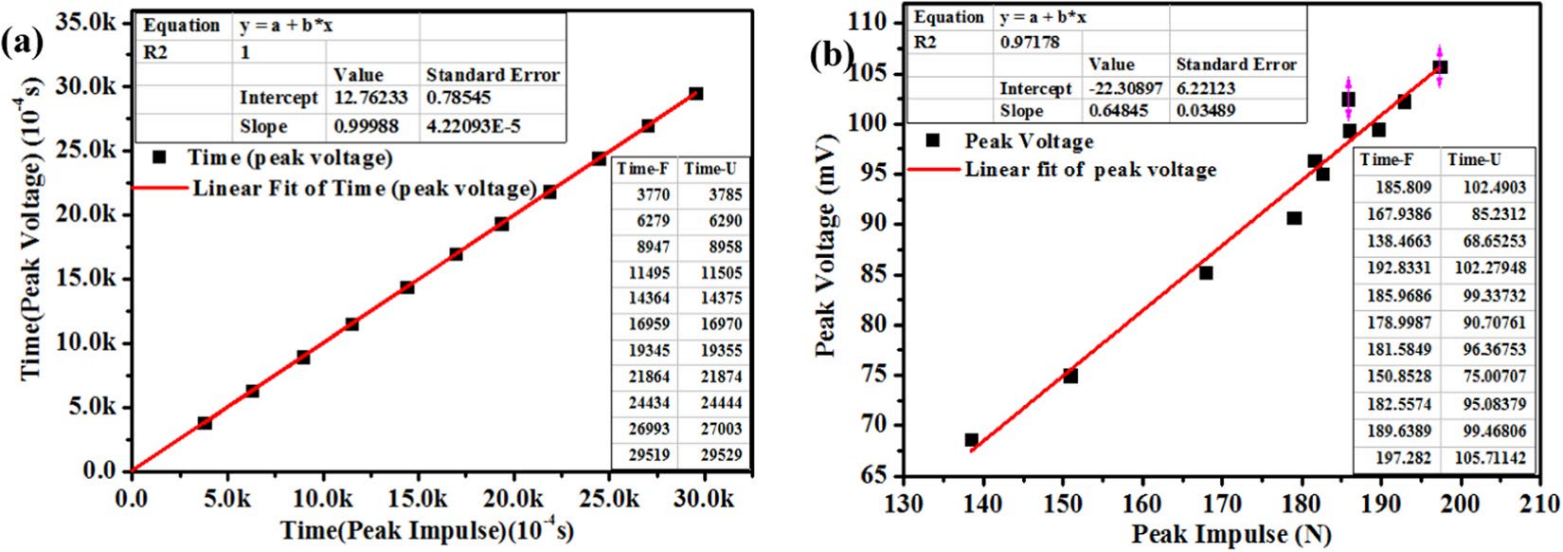
Fitting of the (**a**) peak voltage of the CNT/PVA vs impulse and (**b**) peak voltage vs impulse levels.

Fig. 11 shows the response time, linearity and other parameters of the piezoresistive component of the sensor. The time fitting line is ${y}_{4}=0.99988{x}_{4}+12.76233$ with an *R*^2^ and response time of 1.0 and 1.276 ms, respectively. In Fig. 11b), the voltage fitting line is ${y}_{5}=0.64845{x}_{5}-22.30897$, with an *R*^2^ of 0.97178, and sensitivity of 0.64845. It can be seen from the *R*^2^ values that the peak linearity is appealing; that is, the piezoresistive response capability can meet the dynamic stress/strain detection for SHM applications.

Moreover, as revealed in Table 7, the sensitivities are basically consistent with the sensor requirements. The sensing range of our sensor reaches 0.5 MPa, which is much higher than that shown for other piezoresistive sensors, with most of the full-scale range below 50 kPa. The overall results on piezoelectric/piezoresistive behaviors indicate that our CNT/PVA/ZnO can be a good candidate for attachment to various types of infrastructure for *in situ* monitoring.

**Table 7:** The piezoelectric sensing parameters of the CNT/PVA/ZnO composite under impulse loading for our study and compared to those from other references.

Sensor type	Sensitivity (mV/kPa)	Working potential (V)	Sensing amplitude (Pa)
This work	0.259	10.0	4.93 × 10^5^
Graphene polyurethane^46^	0.26	1.0	9 × 10^3^
SWCNT-PDMS^47^	1.80	2.0	0.6
PDMS/MWCNT^48^	0.23	1.5	5 × 10^4^
Carboxyl-modified fabric^49^	0.585	1.5	—

## Conclusions

Oriented nano-ZnO arrays were successfully grown on a zinc substrate by a hydrothermal process. PVA-doped functionalized CNTs were well self-assembled on the opposite side, and a CNT/PVA/ZnO flexible composite was finally integrated by a spin-coat process to investigate its piezoresistive and piezoelectric performances.

(1) CNT/PVA films with *m*(CNT):*m*(PVA) of 0.2, 0.5, and 1 all present good flexibility. A cost-effective CNT/PVA film with *m*(CNT):*m*(PVA) of 0.5 in tension and compression possesses excellent sensitivity, linear stability, and repeatability with corresponding values of 11.08%/mm and 9.74%/mm, 3.42% and 2.88%, and 2.36% and 2.27%, respectively.

(2) CNT/PVA/ZnO can effectively respond to an impulse load with a high sensitivity, large sensing amplitude, and fast response time of 4.87 mV/lbf, 4.93 × 10^5^ Pa, and 1.496 ms, respectively.

These consequences render CNT/PVA/ZnO flexible composites excellent candidates for piezoresistive and/or piezoelectric sensors in SHM applications.

## Supplementary information

Supplementary information

## References

[1] Schwartz G (2013). Flexible polymer transistors with high pressure sensitivity for application in electronic skin and health monitoring. Nat. Commun..

[2] Gao L (2017). Study on the deformation measurement of the cast-in-place large-diameter pile using fiber bragg grating sensors. Sensor.

[3] Hong CY (2016). Application of FBG sensors for geotechnical health monitoring, a review of sensor design, implementation methods and packaging techniques. Sensor. Actuat. A-Phys.

[4] Li P (2019). Simultaneously identifying displacement and strain flexibility using long-gauge fiber optic sensors. Mech. Syst. Signal Pr.

[5] Buchade PB, Shaligram AD (2007). Influence of fiber geometry on the performance of two-fiber displacement sensor. Sensor. Actuat. A-Phys.

[6] Han S, Chung DDL (2012). Mechanical energy dissipation using carbon fiber polymer-matrix structural composites with filler incorporation. J. Mater. Sci..

[7] Azhari F, Banthia N (2012). Cement-based sensors with carbon fibers and carbon nanotubes for piezoresistive sensing. Cem. Concr. Compos.

[8] Xiao H (2017). Anisotropic electrical and abrasion-sensing properties of cement-based composites containing aligned nickel powder. Cem. Concr. Compos.

[9] Lan C (2018). Improved piezoresistivity of cement-based composites filled with aligned nickel powder. Smart Mater. Struct..

[10] Wang Y (2018). Properties and mechanisms of self-sensing carbon nanofibers/epoxy composites for structural health monitoring. Compos. Struct..

[11] Konstagdoutos MS, Aza CA (2014). Self-sensing carbon nanotube (CNT) and nanofiber (CNF) cementitious composites for real time damage assessment in smart structures. Cem. Concr. Comp.

[12] Luo J (2018). Intrinsic sensing properties of chrysotile fiber reinforced piezoelectric cement-based composites. Sensor.

[13] Mccullagh JJ (2014). Long-term testing of a vibration harvesting system for the structural health monitoring of bridges. Sensor. Actuat. A-Phys.

[14] Chen X (2010). 1.6 V nanogenerator for mechanical energy harvesting using PZT nanofibers. Nano Lett..

[15] Walia S (2015). Flexible metasurfaces and metamaterials: a review of materials and fabrication processes at micro-and nano-scales. Appl. Phys. Rev.

[16] You Z (2016). Design and development of a cutting force sensor based on semi-conductive strain gauge. Sensor. Actuat. A-Phys.

[17] Tian H (2019). A graphene-based resistive pressure sensor with record-high sensitivity in a wide pressure range. Sci. Rep..

[18] Pham GT (2008). Processing and modeling of conductive thermoplastic/carbon nanotube films for strain sensing. Compos. Part B-Eng..

[19] Cai L (2013). Super-stretchable, transparent carbon nanotube-based capacitive strain sensors for human motion detection. Sci. Rep..

[20] Cao CL (2007). Temperature dependent piezoresistive effect of multi-walled carbon nanotube films. Diam. Relat. Mater..

[21] Gau C (2009). Piezoresistive characteristics of MWNT nanocomposites and fabrication as a polymer pressure sensor. Nat. Nanotech.

[22] Alexopoulos ND (2010). Damage detection of glass fiber reinforced composites using embedded PVA–carbon nanotube (CNT) fibers. Compos. Sci. Technol.

[23] Maddipatla, D. *et al*. Development of a novel carbon nanotube based printed and flexible pressure sensor. *IEEE Sens. App. Symp*. Glassboro, NJ, pp. 1–4 (2017).

[24] Loh KJ (2009). Carbon nanotube sensing skins for spatial strain and impact damage identification. J. Nondestruct. Eval..

[25] Zhao MH (2004). Piezoelectric characterization of individual zinc oxide nanobelt probed by piezoresponse force microscope. Nano Lett..

[26] Otal EH (2015). Structural characterization and EXAFS wavelet analysis of Yb doped ZnO by wet chemistry route. J. Alloys Compd..

[27] Bae J (2013). Shim fabrication of vertically aligned ZnO nanocone arrays by wet chemical etching on various substrates and enhanced photoluminescence emission from nanocone arrays compared to nanowire arrays. Phys. Status. Solidi.

[28] Yin YT (2011). Fabrication of ZnO nanorods in one pot via solvothermal method. J. Chin. Chem. Soc..

[29] Parangusan H (2018). Stretchable electrospun PVDFHFP/Co-ZnO nanofibers as piezoelectric nanogenerators. Sci. Rep..

[30] Lipomi DJ (2011). Skin-like pressure and strain sensors based on transparent elastic films of carbon nanotubes. Nat. Nanotechnol..

[31] Xu HY (2004). Hydrothermal synthesis of zinc oxide powders with controllable morphology. Ceram. Int..

[32] Loh KJ (2007). Multifunctional layer-by-layer carbon nanotube-polyelectrolyte thin films for strain and corrosion sensing. Smart Mater. Struct..

[33] Look DC (2005). Evidence for native-defect donors in n-type ZnO. Phys. Rev. Lett..

[34] Tiong TY (2017). A rapid responding ultraviolet sensor based on multi-parallel aligned ZnO nanowires field effect transistor. Sensor. Actuat. A-Phys.

[35] Pauporte, T. Design of solution-grown ZnO nanostructures, toward functional nanomaterials. *Springer* US, (2010).

[36] Naebe M (2008). Electrospun single-walled carbon nanotube/polyvinyl alcohol composite nanofibers:structure–property relationships. Nat. Nanotech.

[37] Alexopoulos ND (2013). Improved strain sensing performance of glass fiber polymer composites with embedded pre-stretched polyvinyl alcohol–carbon nanotube fibers. Carbon.

[38] Wang QG (2016). Graphene “microdrums” on a freestanding perforated thin membrane for high sensitivity MEMS pressure sensors. Nanoscale..

[39] Li H (2006). Effect of compressive strain on electrical resistivity of carbon black-filled cement-based composites. Cem. Concr. Comp.

[40] Vertuccio L (2015). Strain and damage monitoring in carbon-nanotube-based composite under cyclic strain. Compos. Part A-Appl. Sci.

[41] Su CC (2012). Two dimensional carbon nanotube based strain sensor. Sensor. Actuat. A-Phys.

[42] Zegeye E (2013). The strain sensing property of carbon nanofiber/glass microballoon epoxy nanocomposite. Smart Mater. Struct..

[43] Rajpure KY, Bhosale CH (2000). Photo)electrochemical investigations on spray deposited n-Sb_2_S_3_ thin film/polyiodide/photoelectrochemical solar cells. Mater. Chem. Phys..

[44] He HX (2018). Optimal sensor placement for spatial structure based on importance coefficient and randomness. Shock Vib.

[45] Outline/installation drawing model series 1203v force sensors, https://www.dytran.com/images/product_pdf/1203V1_ds.pdf.

[46] Yao HB (2013). A flexible and highly pressure-sensitive graphene-polyurethane sponge based on fractured microstructure design. Adv. Mater..

[47] Wang X (2014). Electronic skin: silk-molded flexible, ultrasensitive, and highly stable electronic skin for monitoring human physiological signals. Adv. Mater..

[48] Lipomi DJ (2011). Skin-like pressure and strain sensors based on transparent elastic films of carbon nanotubes. Nat. Nanotech.

[49] Kwon D (2016). Highly sensitive, flexible, and wearable pressure sensor based on a giant piezocapacitive effect of three-dimensional microporous elastomeric dielectric layer. ACS Appl. Mater. Inter.

