# Supplementary material for: Fabrication and Piezoresistive/Piezoelectric Sensing Characteristics of Carbon Nanotube/PVA/Nano-ZnO Flexible Composite
Source: Sci Rep. 2020 Jun 1;10:8895. doi: 10.1038/s41598-020-65771-x (PMC7264348; doi:10.1038/s41598-020-65771-x)
Supplement: Supplementary file 1 — Supplementary information [file 41598_2020_65771_MOESM1_ESM.docx]

**Supplementary Information**

**2. Experimental details**

*2.1. Raw Materials*

**Table S1.** Main physical properties of the CNTs.

| Diameter (D/nm) | Length (L/μm) | Purity (wt.%) | Density (g/cm^3^) | BET surface area (m^2^/g) | Electrical conductivity (S/cm) |
| --- | --- | --- | --- | --- | --- |
| 10-20 | 10-15 | ≥95 | 2.1 | 40-300 | ≥300 |

**Table S2.** Main properties and the corresponding providers of the reagents.

| Reagent name | Content (wt%) | Density (g/ml) | Quality | Provider |
| --- | --- | --- | --- | --- |
| Sulfuric acid | 98 | 1.84 | AR | Sinopharm Co. Ltd., CN |
| Nitric acid | 70 | 1.42 | AR | Sinopharm Co. Ltd., CN |
| Hydrochloric acid (HCl) | 37 | 1.477 | AR | Sinopharm Co. Ltd., CN |
| Ethanol | 99.5 | 0.789 | AR | Sinopharm Co. Ltd., CN |
| Distilled water (DSW) |  | 1.0 |  | Lab homemade, CN |
| Nickel chloride |  | 3.55 | AR | Sinopharm Co. Ltd., CN |
| Toluene |  | 0.87 | AR | Shanghai Aier Chemical Agent Co. Ltd., CN |
| Tetrahydrofuran (THF) |  | 0.89 | AR | Fuyu Reagent Co. Ltd., CN |
| Polyvinyl alcohol (PVA) | 98 | 1.3 | AR | Sinopharm Co. Ltd., CN |
| Azobisisobutyronitrile (AIBN) |  | 1.1 | AR | Tianjin Daimao Chemical Reagent Factory, CN |
| Conductive silver |  |  |  | Shanghai Synthetic Resin Research Institute, CN |
| Zinc flake | 99 | 7.14 | AR | Tianjin Fuchen Chemical Reagents Factory, CN |
| Zinc acetate | 99 | 1.84 | AR | Sinopharm Co. Ltd., CN |
| Ammonia | 25-28 | 0.91 | AR | Sinopharm Co. Ltd., CN |

*2.5. Characterization methods*

The piezoresistive, and piezoelectric sensing characteristics of the CNT/PVA/ZnO flexible composite were tested by cyclic three-point bending loads and impulse load testing methods, respectively, and the details are demonstrated as follow.

**Cyclic three-point bending loads test**― A universal testing machine (CMT5205/5305 type, MTS Industrial Systems (China) Co., Ltd., China) in tandem with a DC circuit consisting of a DC power supply (the applied voltage setting as 2 V), wires, and two digital multimeters (VC9808+ type, VICTOR, China) were employed to collect the applied loads and resistance of the CNT/PVA film and CNT/PVA/ZnO film that was strongly adhered to a flexible PP plate (Fig. S1a). The load was cyclically loaded in mid-span displacement control mode at 2.0 mm/min, and the triangular wave loading was adopted for the mid-span displacement control mode from 0 mm to 5 mm and repeated 5 times (Fig. S1b).

| 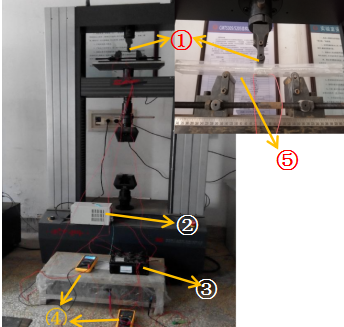  (a) | **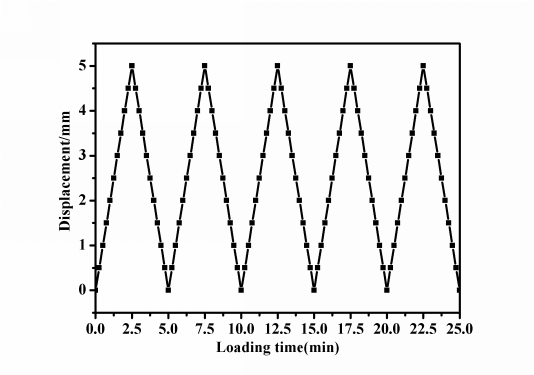**  (b) | | |
| --- | --- | --- | --- |
| **Figure S1.** (a) Apparatus used for piezoresistive testing of CNT/PVA/ZnO under three-point bending and (b) cyclic loading mode results (① universal testing machine, ② DC power, ③ standard resistor, ④ digital multimeter, and ⑤ PP plate substrate). | | | |
| 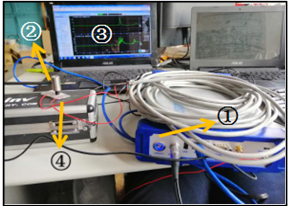  (a) | | 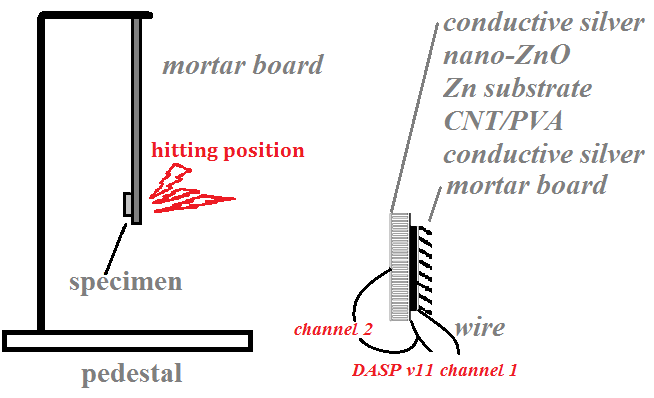  (b) |  |
| **Figure S2.** (a) Data acquisition system for CNT/PVA/ZnO testing under impulse loading (① dynamic data collector, ② electronic hammer, ③ laptop, ④ CNT/PVA/ZnO) and (b) schematic of fixture for CNT/PVA/ZnO sheet. | | |  |

**Impulse load test**―The CNT/PVA film side of the flexible composite was adhered onto the free end of a mortar board with a size of 400 mm×100 mm×20 mm, while the nano-ZnO component of the flexible composite was exposed. A simple fixture device was used to fix the other end of the mortar board to form a cantilever (detailed as Fig. S2). A dynamic data acquisition system (DASP-V11 type, Orient Institute of Noise & Vibration, Beijing, China) collected the applied impulse loads and electric responses of CNT/PVA/ZnO sheet against time under cyclic impulse loading exerted by a hammer at a 2 N/s head speed. To precisely observe the response hysteresis of the sensor owing to the introduction of PVA, a high sampling frequency of 10240 Hz was adopted, the sampling period was 3 s, and the frequency was multiplied by 3 s to obtain 30720 sampling points.
